# Towards equality: gender representation at the Royal College of Radiologists’ Annual Scientific Meeting 2014-2021

**DOI:** 10.12688/wellcomeopenres.18439.1

**Published:** 2022-12-05

**Authors:** Lorna M. Gibson, Kayleigh L. Wood, Joanna M. Wardlaw

**Affiliations:** 1 Centre for Biomedicine, Self and Society, Usher Institute, University of Edinburgh, Edinburgh, UK; 2 Department of Clinical Radiology, New Royal Infirmary of Edinburgh, Edinburgh, UK; 3 Centre for Clinical Brain Sciences, Edinburgh Imaging, and Dementia Research Centre, University of Edinburgh, Edinburgh, UK

**Keywords:** Gender equality, inclusion, diversity, female, male, radiologists, conference, career

## Abstract

**Background: **Conferences facilitate career advancement, but gender imbalances in public fora may negatively impact both women and men, and society. We aimed to describe the gender distribution of presenters at the UK’s 2014-2021 Royal College of Radiologists’ (RCR) Annual Scientific Meeting.

**Methods:** We extracted data on presenter name, role and session type from meeting programmes. We classified gender as male or female using names, records or personal pronouns, accepting the limitations of these categories. We classified roles by prestige: lead, other (speakers and workshop faculty), proffered paper or poster presenters. We calculated odds ratios (OR) and 95% confidence intervals (CI) for associations between gender and binary outcomes using logistic regression.

**Results: **Women held 1,059 (37.5%) of 2,826 conference roles and presented 9/27 keynotes. Compared to men, women were less likely to hold other roles such as speakers and workshop faculty (OR 0.72 95% CI 0.61-0.83), and more likely to present posters (OR 1.49 95% CI 1.27-1.76). There were 60 male-only and eight women-only multi-presenter sessions. Sessions led by women had higher proportions of women speakers. The odds of roles being held by women increased during online meetings during COVID (OR 1.61, 95% CI 1.36-1.91).

**Conclusion: **The proportion of women presenters and keynote speakers reflects that of RCR membership, but not of the patient population. Disadvantage starts from the earliest career stages, prejudicing career opportunities. Efforts to improve inclusion and diversity are needed; focusing on lead roles and hybrid online/in-person formats may accelerate change.

## Introduction

Studies of science, academia and business show that diversity is positively associated with improved productivity, effectiveness, communication and innovation, and the benefits of a diverse workforce are increasingly recognised within healthcare ^1, 2^. Diversity can improve the quality of care, employees’ job satisfaction, and the impact of research and collaboration, and reduce risk taking ^1, 2^. 

Unfortunately gender imbalances persist within medicine - globally and at all levels of, and disciplines within, the profession ^2^. Systemic, organisational, cultural and individual factors differentially impact women compared to men, limiting women’s career progression ^2, 3^. Female doctors are under-represented in senior academic roles, medical society leadership positions and journal editorial boards ^3– 11^. Female doctors in UK hospitals earn an average of 19% less than male doctors, a pay gap partly accounted for by fewer women holding the highest paid positions ^12^. A higher proportion of women doctors work less-than-full-time to accommodate their disproportionately higher burden of unpaid care work ^12– 14^. Inflexible working pattern policies and maternity and parental leave disproportionately impact on the daily work and the career progression of women, and reinforce pervasive stereotypes of both women and men at work ^3, 15^. Such stereotypes can manifest materially, with women reporting limitations on their training opportunities, and men reporting pressure to prioritise work over family life ^15– 17^, impacts that ultimately further entrench gender inequality and impede necessary change.

Conference speakers have the opportunity to share knowledge, express views, develop networks and gain visibility and professional recognition ^18– 22^. Delivering prestigious keynote presentations and chairing sessions can strengthen applications for promotion and research funding at mid and senior levels ^20– 22^ while oral presentations increase visibility, research impact, and chances of a good start to career advancement at junior levels. As women are under-represented as invited speakers at medical speciality conferences, they have fewer opportunities to reap these benefits ^19, 22, 23^, or to benefit the community at large: diverse conference speakers broaden perspectives and ideas, and provide a broader variety of role models to inspire trainees and medical students ^18, 23^.

Efforts to address gender disparities in radiology, such as the Women in Focus initiative at the European Society of Radiologists’ meeting 2019 ^24^, the American Association of Women Radiologists ^25^ and Women in Interventional Radiology ^26^, have set out programmes, monitoring strategies and statements to support the underlying principles of equality, diversity and inclusion. These organisations and others provide networks, workshops and mentorship to support the development, visibility and success of women ^25, 27, 28^, and have lobbied for changes to pregnancy and parental leave policies ^25^. Conference organisers have responsibilities to encourage diverse participation, whilst compiling a programme of engaging and interesting speakers, including field experts and covering a variety of novel topics and quality content ^22^. The development of hybrid online and in-person conferences as a result of the COVID-19 pandemic may improve access to conferences for potential speakers and audience members and reduce the environmental impact of these events.

Evidence on patterns of gender representation at radiology conferences is required to inform policies and provide measures to benchmark progress. Using data from the 2014–2021 Royal College of Radiologists’ Annual Scientific Meeting (RCRASM), this study aimed to describe the patterns of the participation of women and men in conference roles of varying levels of prestige.

## Methods

### Ethics

This study was deemed to not require ethics approval, as it makes use of data already within the public domain. Ethics approval was waived by ACCORD at the University of Edinburgh, UK.

The RCRASM is a yearly event held in the United Kingdom, comprising of keynote presentations, lectures and workshops on clinical topics, and opportunities to present research and audit orally and in poster form. In 2020 and 2021, the RCRASM was held online due to the COVID-19 pandemic.

### Data extraction

One author (LMG) extracted data from all the Royal College of Radiologists (RCR) annual scientific meeting (ASM) full conference programmes that were available online, covering the period 2014–2021. For each session, we extracted all data on presenters’ names, and classified their role and the session type (and repeated this for presenters who were involved in multiple roles within the same session, for example chairing and lecturing). We extracted the names of the Scientific Committee, the Ansell poster reviewers, the audit poster reviewers and the audit poster prize judges. We contacted the RCRASM administrative committee if this information was not publicly available, and for names/genders of presenters listed as ‘to be confirmed.’

***Classification of gender, conference session type and presenters’ roles.*** Using presenters’ names, one author (LMG) classified gender as either female or male, and confirmed the classification using the General Medical Council (GMC) Register of Medical Practitioners, or photographs or text containing personal pronouns from institutional or the RCR websites for non-GMC-registered presenters, accepting that some presenters may not identify with these binary gender categories. If only a presenter’s first initials were provided, we checked the RCR website for presentation materials to identify their first name. If this could not be found, but a GMC record matching the first initial and surname with a training programme listed as clinical radiology or clinical oncology, or specialist registration as a clinical radiologist or clinical oncologist was found with no other potential matching record for a doctor of another gender or specialty, we classified gender according to the available GMC record, assuming a match. If we could still not find information on the presenter’s first name, we checked any available LinkedIn, Instagram and Facebook profiles and correlated professional titles and workplaces. If we could still not find information on full names to allow us to classify gender, we emailed the RCR.

One author (LMG) classified session types using the conference programme titles (as either lecture, workshop, keynote, proffered papers, poster presentations [either ePoster, scientific, audit or pictorial review poster presentations], or other panel debate, lunchtime symposium, quiz or interactive discrepancy meeting or case based discussion, or Schwartz round).

We categorised presenters’ roles according to four levels of prestige: lead roles (lecture session chair, workshop leader, keynote speaker, lunchtime symposium chairs and speakers, panel chair, quiz session chair or Schwartz round facilitator); other roles (lecture session speaker, workshop faculty, quiz panellist, panel participant, interactive discrepancy meeting participant, interactive case study participant, Schwartz round participant); oral presenters of proffered papers; or poster presenters.

If sessions continued over multiple time slots in the programme (e.g. before and after a coffee break), we counted chairs of these sessions once, unless the programme indicated that the chairperson changed. If the same person acted as both chair and a named lecturer of a titled presentation within the same session, or chaired a panel debate and presented as a panellist, or led a workshop and presented a titled talk during a workshop, we counted these people as having presented twice and extracted data on both of their roles. During 2020 and 2021, all sessions were online, due to the COVID-19 pandemic, and ‘case-based interactive sessions’ were classed as lectures only if titled talks were listed. If a session contained a panel discussion with panellists described as ‘all faculty,’ or named participants, we counted each presenter as also participating in a panel discussion. If a programme listed a ‘discussion’ or ‘question and answer session’ presenters were not counted twice, as these elements of a session are usually standard. If workshops were repeated by the same presenters during a conference, we counted these presentations twice. We excluded industry-led workshops presented solely by non-NHS presenters, as our paper focuses on opportunities for radiologists at the RCR ASM. A 10% subset of conference materials were double read by a second author (KLW) to check accuracy of data extraction and differences were resolved by discussion.

### Statistical analyses

We present descriptive data for each of the eight included RCRASM conferences (2014–2021) on the proportions of the different conference roles which were presented by women and men, the numbers of women and men who held one versus more than one role, and the numbers of sessions with no women and those with all women presenters.

We calculated odds ratios (OR) and 95% confidence intervals (CI) to assess for associations between gender and binary outcomes using logistic regression. We used SPSS (version 27) for analyses, and Excel 2013 for data display.

## Results

**Figure 1.**
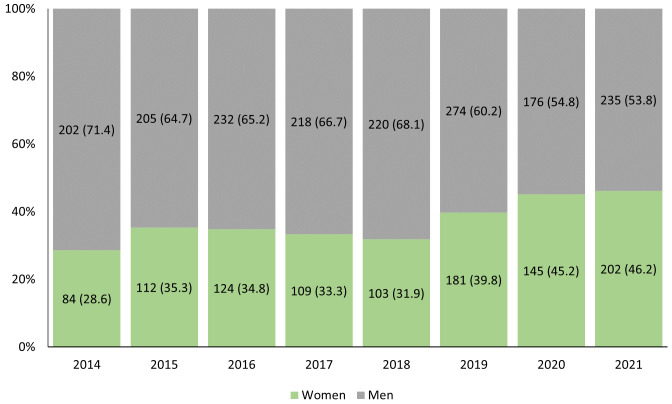
N (%) roles held by women and men at each RCRASM conference from 2014–2021.

We were able to classify the gender for over 99% of presenters, for whom the RCRASM provided 2,826 opportunities to fill roles between 2014-2021, of which 1,059 (37.5%) were held by women and 1,767 (62.5%) by men ( Figure 1) ^29^. On double checking a sample of 10% of records, two authors were in agreement on all presenters’ gender classifications and on 99% of their conference role classifications.

The proportions of roles filled by women has increased from 2014–2021, although most presenters each year have been men ( Figure 1). In 2020 and 2021, the RCRASM was held online due to the COVID-19 pandemic. Compared to pre-pandemic years, the odds of a role being filled by a woman increased during the pandemic (OR 1.61, 95% CI 1.36-1.91). 

### Conference roles

Fewer lead roles were filled by women than men between 2014–2021 (n=156 versus n=270, Table 1), although there were no significant differences in the proportions of women and men who held lead roles, or who presented proffered papers ( Table 1). Compared to men, women were less likely to hold other roles such as speakers and workshop faculty (OR 0.72 95% CI 0.61-0.83), and more likely to present posters (OR 1.49 95% CI 1.27-1.76) ( Table 1).

**Table 1.** Conference roles filled by women and by men in the RCR ASM 2014–2021.

Role	Roles filled by women n (%) N=1,059	Roles filled by men n (%) N=1,767	OR (95% CI) for roles filled by women compared to men
Lead role	156 (14.7)	270 (15.3)	0.96 (0.77-1.19)
Other role ^1^	500 (47.2)	982 (55.6)	0.72 (0.61-0.83)
Proffered paper presenter	39 (3.7)	56 (3.2)	1.17 (0.77-1.77)
Poster presenter	364 (34.4)	459 (26.0)	1.49 (1.27-1.76)

1 Other role includes: lecture session speaker, workshop faculty, quiz panellist, panel participant, interactive discrepancy meeting participant, interactive case study participant, Schwartz round participant

**Figure 2.**
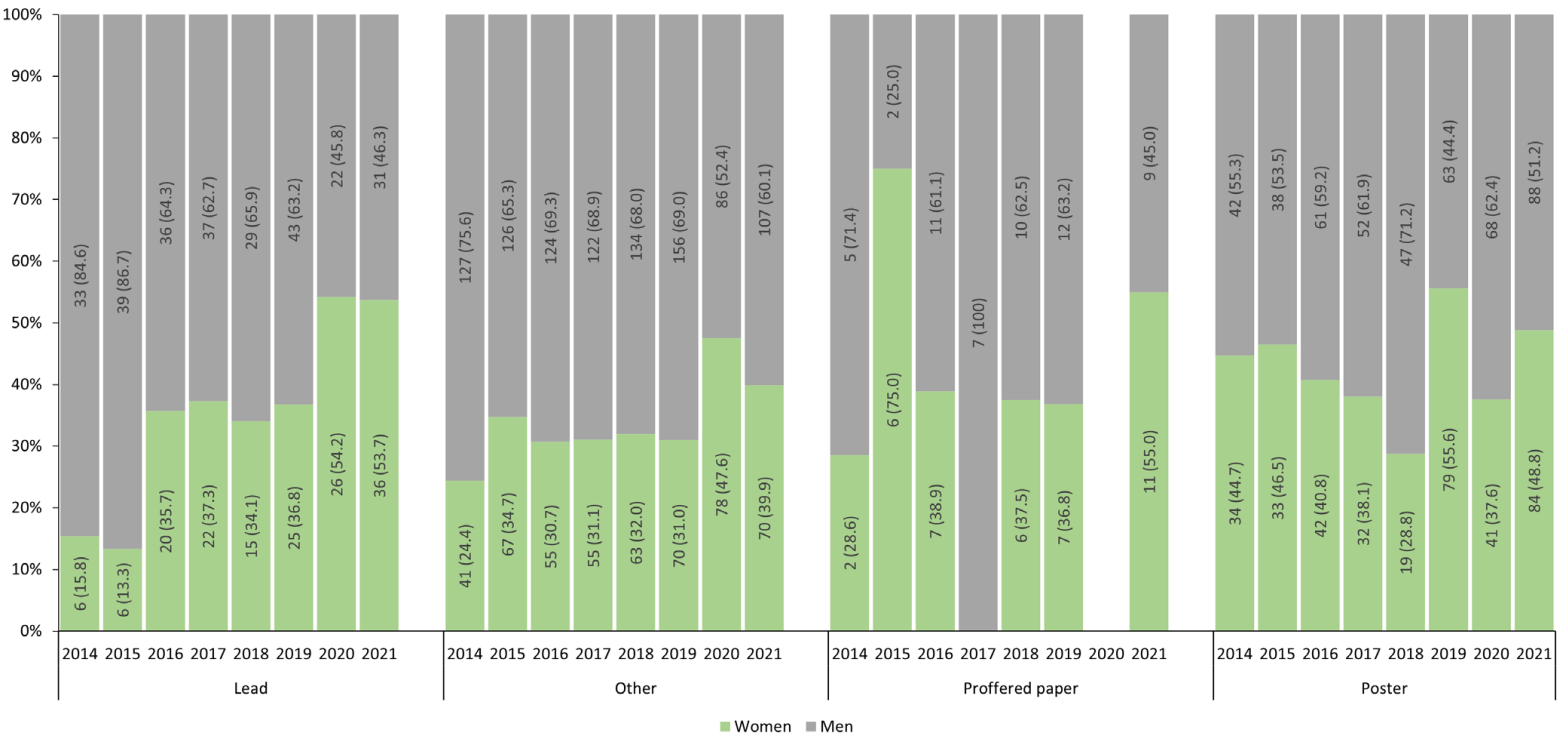
Proportions of prestigious and less prestigious roles presented by women and men at RCRASM 2014–2021.

From 2014 to 2021, the proportions of lead roles held by women has increased, with women filling just over half of lead roles during the online conferences of 2020 and 2021 ( Figure 2). The proportions of other roles filled by women also increased during 2020 and 2021, as did the proportions of women presenting proffered papers in 2021 ( Figure 2).

### All-male and all-female sessions

**Figure 3.**
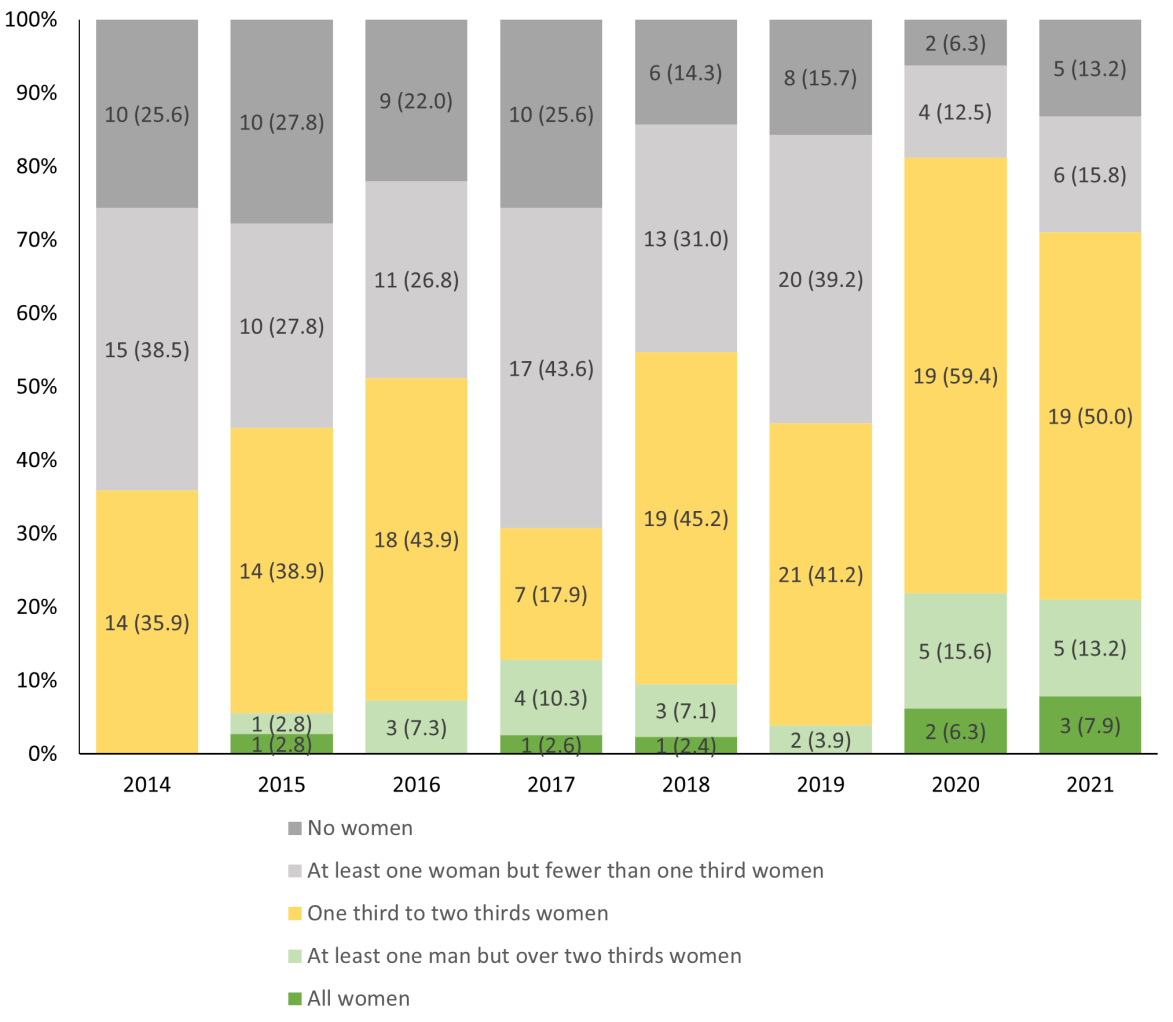
N (%) sessions comprising all women, no women and mixed-gender groups of presenters.

Between 2014 and 2021, there were 318 multi-presenter lecture, workshop, panel debates, quiz sessions, lunchtime symposia, interactive discrepancy meetings or Schwartz rounds, which included at least two (and up to 18) presenters. Of these sessions, 60/318 (18.9%) consisted only of male presenters, and 8/318 (2.5%) consisted of only female presenters ( Figure 3, Table 2). Women constituted the vast majority of participants in up to 22% of sessions per year. During the online conferences, larger proportions of multi-presenter sessions had a balance of male and female speakers ( Figure 3).

**Table 2.** Involvement of women in RCR ASM 2014–2021 by multi-presenter lecture, workshop or panel session topic.

	Number of sessions involving:					
Topic	No women presenters	At least one woman but less than one-third of presenters were women	One-third to two-thirds of presenters were women	At least one man but over two-thirds of presenters were women	All women presenters	Total N sessions per topic
Neonatal and fetal	0	0	2	1	1	4
Breast, gynaecology, pregnancy	0	2	2	6	3	13
Uroradiology *	0	3	3	1	0	7
Radiotherapy	0	3	12	4	1	20
Academia	1	7	14	1	0	23
Chest	4	4	4	2	0	14
Professional issues	9	3	11	4	1	28
Other	2	2	4	1	0	9
Education and teaching	0	0	5	0	2	7
Trauma	0	0	1	0	0	1
General oncology	0	4	9	0	0	13
General radiology	4	11	15	1	0	31
Trainees or on-call	5	8	13	0	0	26
Nuclear medicine	1	3	3	0	0	7
Paediatrics	3	3	5	0	0	11
Neuro head and neck	5	10	4	2	0	21
Musculoskeletal	6	12	4	0	0	22
Cardiac	3	6	3	0	0	12
Gastrointestinal and HPB	5	6	6	0	0	17
Intervention and vascular	7	7	1	0	0	15
Prostate	4	1	2	0	0	7
Global health	1	0	5	0	0	6
Artificial intelligence	0	1	3	0	0	4
**Total**	**60**	**96**	**131**	**23**	**8**	**318**

HPB = hepatobiliary *Involving topics relevant to patients of any gender

The proportions of women involved in lecture, workshop or panel sessions varied by topic of the session ( Table 2). Women made up the majority of presenters in at least one session on neonatal and fetal imaging, breast/gynaecology/pregnancy, uroradiology, radiotherapy, academia, chest radiology, professional issues and education and teaching during the RCR ASM from 2014–2021 ( Table 1). Of the 15 interventional radiology sessions, women made up one-third to two-thirds of presenters in only one session ( Table 2).

### Lecture and workshop sessions with and without women leaders

Of the 281 lecture and workshop sessions held between 2014 and 2021, compared to sessions that did not involve any women leaders, in sessions that were led by at least one woman, women more frequently accounted for higher proportions of presenters ( Table 3).

**Table 3.** Proportions of women presenters in lectures and workshops led by at least one or no women.

Proportions of women presenters	N (%) sessions with no woman leader (N=177)	N (%) sessions with at least one woman leader (N=104)
No women	59 (33.3)	18 (17.3)
At least one woman, but less than one third women	43 (24.3)	26 (25.0)
One third to two-thirds women	63 (35.6)	44 (42.3)
At least one man, but more than two-thirds women	7 (4.0)	9 (8.7)
All women	5 (2.8)	7 (6.7)

### Keynote speakers

Between 2014 and 2021, there were 27 keynote speakers, of whom a third were women (9/27) and two-thirds were men (18/27). In 2014, 2015 and 2021 all keynote presentations were delivered by men, and in 2020 the single keynote presentation was delivered by a woman.

### Conference committees

Details of organising committees were listed in RCRASM programmes from 2014–2016. From the available data, the scientific committees, and scientific abstract reviewers were predominantly men ( Table 4). In contrast, groups of audit abstract reviewers and judges tended to be more equally representative of men and women ( Table 4). Men were in the minority in only one committee over these three years: audit poster prize judges in 2016 ( Table 4).

**Table 4.** Women and men involved in committees and as reviewers.

	Year	N (%) women	N (%) men
Scientific committee	2014	2 (15.4)	11 (84.6)
	2015	2 (18.2)	9 (81.8)
CR Professional learning and development committee	2016	4 (26.7)	11 (73.3)
CO Professional learning and development committee	2016	3 (50.0)	3 (50.0)
Scientific poster abstract reviewers	2014	5 (23.8)	16 (76.2)
	2015	4 (13.8)	25 (86.2)
	2016	5 (29.4)	12 (70.6)
Audit poster abstract reviewers	2014	5 (38.5)	8 (61.5)
	2015	6 (50.0)	6 (50.0)
	2016	4 (50.0)	4 (50.0)
Audit poster prize judges	2014	0 (0)	3 (100)
	2015	2 (50.0)	2 (50.0)
	2016	2 (66.7)	1 (33.3)

CR = clinical radiology, CO = clinical oncology

## Discussion

### Main findings

Women held 37.5% of conference roles and presented one-third of keynotes during the 2014–2021 RCRASM. Women were also less likely than men to hold mid-prestige roles such as speakers or workshop faculty, and much more likely to hold low-prestige poster presenter roles. Male-only sessions are over seven times more common than female-only sessions, and scientific committees comprised predominantly of men. However, women’s inclusion in the RCRASM increased during 2020–2021 (when the conference was online due to the COVID-19 pandemic) and when prestigious chair and workshop leadership roles were held by women, higher proportions of women held the mid-prestige speaker and workshop faculty roles. Apart from these two latter exceptions, the overall pattern of over-representation in posters and under-representation in keynotes, chairs and invited speakers simply reinforces the low prestige/low visibility roles occupied by women, perpetuating career disadvantage and other adverse effects.

### Comparison with other studies

The proportion of women radiologists involved in conferences does not represent the population from which our profession is recruited or the patients we serve. The proportion of women involved in the RCRASM from 2014–2021 reflects that of RCR consultant membership (37%, ^30^), and is similar to the proportions of women presenters at the 2018 Radiological Society of North America conference (RSNA) (35% ^31^), and at the Association of University Radiologists’ (AUR) and the American Roentgen Ray Society (ARRS) conferences in 2009, 2014 and 2019 (39%, ^32^).

Women conference participants are overrepresented in low-prestige roles, and under-represented in high-prestige roles, inadvertently creating programmes of male ‘killer’ and female ‘filler.’ Similar to our findings, 35% of keynote presentations were presented by women during three ARRS conferences ^32^. Women surgeons are over-represented in non-technical presentations, more likely to introduce speakers and present awards, and less likely to give technical and scientific presentations ^33, 34^.

Our study and others suggest a recent trend towards improved gender balance in conferences although this trend varies across different conferences and time periods ^19, 22, 23, 35^ and may have been accelerated artificially by the COVID-19 pandemic. The increase in women presenters in the RCRASM during 2020 and 2021 may relate in part to the online format necessitated by the COVID-19 pandemic; it remains to be seen whether these changes are sustained and therefore are likely to be truly reflective of broader cultural changes in attitudes toward gender diversity over recent years. Online conferences facilitate attendance through reducing barriers relating to time, travel and expenses, and may improve participation by people from diverse means and backgrounds, such as those on lower incomes and with unpaid care responsibilities ^36– 38^. Two of five critical care conferences held between 2013–2017 showed significantly increased female representation this period ^39^ and while there was no temporal trend in gender distribution at multiple rheumatology conferences between 2015–2019 there was a narrowing of the gender gap compared to 2003 and 2004 ^40^.

All-male panels are much more common than all-female panels in the RCRASM and other clinical conferences, and the gender balance of those in conference leadership positions reflects that in less prestigious roles. During 2017–2018, 37% of panels in medical conferences were all-male compared to 7% all-female ^41^, and 40% of surgical conference sessions are all-male. All-male panels are more common when organised by all-male groups of conveners ^42^. In surgical society meetings, having at least one female convener was significantly associated with fewer all-male panels ^23^, as was the presence of women in conference leadership roles ^43^, and sessions with at least one female co-ordinator are significantly associated with a higher proportion of female presenters compared to those with all-male coordinators e.g. 36% vs 7% female presenters ^44^.

### Strengths and limitations

Previous studies have focused on a single year of a conference ^31, 45^, or focused on subgroups of conference presenters ^45^ or presenters only without assessing the gender balance of conference organising committees ^19, 31, 32, 40, 45^. In contrast, our study makes use of eight years of data from the RCRASM, using information on presenters in all roles, and on the gender balance of the conference committees to give as complete a picture as possible of the patterns of involvement of women and men at all levels of the RCRASM, and to assess for changes in these patterns over recent years. Double checking of data showed high (>99%) agreement on the classification of gender and conference role, indicative of a reliable dataset.

We were not able to classify the gender of 14/2,747 (0.5%) presenters, due to first names being listed as initials, although such a small portion is unlikely to have affected our main results. We also acknowledge that our binary classification of gender in this study limits our ability to inform on the representation of people with non-binary gender identities within the RCRASM, or on any other form of imbalance relating to a protected characteristic. As we focussed are analyses on the roles and the gender of the person who filled them (rather than on individual presenters), our data are not affected by presenters who changed their name or submitted works under different variants of their names.

We used publicly available conference programmes to assess gender balance, and in doing so we are unable to comment on further sources and manifestations of gender imbalance. Reasons for under-representation of women at conferences can be in part due to female speakers declining invitations ^21^ and women may opt for shorter presentations and posters compared to male counterparts ^20^. Data on women and men who were invited to participate and declined (or who submitted oral or poster abstracts and were rejected) are not publicly available (but should be made so yearly by the RCR), and as we were unable to attend all of the conferences (and not all sessions are recorded) we are unable to comment on differences in speaking time, formality of introduction of male and female speakers by male and female chairs, or the numbers and genders of audience members asking questions. During AUR 2009, women gave significantly shorter (mean of 5.7 minutes) presentations and disproportionately fewer women than men gave presentations longer than 30 minutes, but this improved in subsequent years ^32^. In RSNA 2018, women asked questions in only 24% of sessions and when they did participate, they spoke for a mean of 7 seconds, compared to 29 seconds for men ^31^. Although our study does not capture such metrics of participation, doing so at future RCRASMs would round out our knowledge of the gender balance at the conference.

### Implications for patients, doctors, researchers and policy

Several conferences have shown improved gender balance following new policy implementation. The Society of Interventional Radiologists (SIR) required prospectively identified women to be invited as speakers at the SIR ASM, resulting in an increased proportion of female presentations, from 9% in 2016 to 14% in 2018 ^44^, and dedicated recruitment efforts to attract and encourage female trainees are being made ^46^. Women gave 48.5% of oral presentations at the 2015 American Society for Microbiology General Meeting, after organising committees were made aware of data on gender balance, and increased the numbers of women involved in convening sessions and deliberately avoided convening all-male panels ^47^. Although evidence of association cannot be taken as evidence of causation, these pragmatic strategies could be implemented by the RCRASM and other conference committees, and have the potential to swiftly improve gender balances in presenters, efforts that are most needed where greater gender imbalance exists, such as in interventional radiology ^28, 44, 48^. However it should be noted that conference planning should take account of the higher attrition rate of female invitees at all stages in the process. For example, the European Stroke Organisation Conference increased female faculty from <33% in 2019 to 43% in 2022 through positive action; however, despite starting with a 50:50 female to male invite list by 2022, women invitees were far more likely to decline, or accept but then have to withdraw, due to domestic or work commitments, resulting in <50% presence in the end (JMW personal communication).

The 2022 RCRASM rejected an abstract of this current work. Although many support the idea of gender balance in theory, this does not inevitably lead to a change in practice ^49^ and consistent efforts from both within and external to conference organisations is needed to bring about meaningful change. Publicly available data from ongoing monitoring of gender equality would hold organisations to account, and inform real-time policy-making to create genuine change. Our study and the references herein describe a number of metrics that could be used. Priority targets would be those areas in which improvement is needed most rapidly and would have the most impact: the most prestigious roles of session leadership and keynote speakers.

However, implementing policies that result in more women in positions traditionally dominated by men fails to challenge our ideas of gendered norms relating to work and domestic lives, or the complex relationships of conferences and profit-seeking industry, or the impacts of conference attendance on environmental sustainability. Men who say no to work may experience stigma, with male doctors who request to work less-than-full-time reported they were made to feel ‘guilty and weak,’ and lacking commitment ^12^. In clinical couples, female doctors are significantly more likely to bear the majority of unpaid domestic work ^13, 14^, with 29% of women describing their career progression being limited by their male partner’s job, as opposed to 15% of men ^12^.

Failing to challenge gender disparity in conferences limits career opportunities for women from the earliest stages, pressurises men to participate in work at the expense of caring roles, and reinforces gender-based norms around the distribution of paid and unpaid domestic labour with material and social consequences for all. Hybrid conferences would not only reduce the environmental impact of these events but also enable all – men, women and those identifying as non-binary – the flexibility to manage their paid and unpaid work. Progressive conference programming policies informed by publicly available data and targeted at gender representation within the most prestigious roles are needed to hold organisers to account and accelerate change.

## Data Availability

RCRASM conference programmes were made publicly available online each year by the Royal College of Radiologists, and readers are directed to enquiries@rcr.ac.uk for access. Edinburgh Datashare: Towards equality: gender representation at the Royal College of Radiologists’ Annual Scientific Meeting 2014-2021 https://doi.org/10.7488/ds/3776 ^29^ This project contains the following underlying data: 220413_Full_programme_dataset_datashare.sav Data are available under the terms of the Creative Commons Attribution 4.0 International license (CC-BY 4.0).
