# Supplementary material for: Towards equality: gender representation at the Royal College of Radiologists’ Annual Scientific Meeting 2014-2021
Source: Wellcome Open Res. 2023 Jul 24;7:291. Originally published 2022 Dec 5. [Version 2] doi: 10.12688/wellcomeopenres.18439.2 (PMC10422055; doi:10.12688/wellcomeopenres.18439.2)
Supplement: Peer review history

# Peer review: Reviewer response for version 2

**DOI:** 10.21956/wellcomeopenres.21896.r63635

**Published:** 2023-08-11

**Authors:** Christine Kremer

**Affiliations:** 1 Neurology Department, Skåne University Hospital, Department of Clinical Sciences, Lund University, Malmö, Sweden

Thank you for the thorough revision. I have no further comments.

Is the work clearly and accurately presented and does it cite the current literature?

Yes

If applicable, is the statistical analysis and its interpretation appropriate?

Yes

Are all the source data underlying the results available to ensure full reproducibility?

Yes

Is the study design appropriate and is the work technically sound?

Yes

Are the conclusions drawn adequately supported by the results?

Yes

Are sufficient details of methods and analysis provided to allow replication by others?

Yes

Reviewer Expertise:

Sex and gender differences in stroke.

I confirm that I have read this submission and believe that I have an appropriate level of expertise to confirm that it is of an acceptable scientific standard.

# Peer review: Reviewer response for version 1

**DOI:** 10.21956/wellcomeopenres.20448.r55357

**Published:** 2023-03-21

**Authors:** Rachel Palmen

**Affiliations:** 1 Open University of Catalonia, Barcelona, Spain

This article provides an analysis of empirical data of gender representation at the Royal College of Radiologists- Annual Scientific Meeting 2014/2021. The use of data from 2014-2021 provides a timespan that is sufficiently extensive to frame a convincing argument about the uneven representation of women in different conference roles. It successfully contextualises the data- as results show it is not representative of the " *population from which our profession is recruited or the patients we serve*"… yet does " *reflect RCR consultant membership*" (37%) - which is an important consideration.

 It also provides a brief reflection on the implications of gendered inequalities in conference participation particularly in terms of “cumulative disadvantage”, i.e. how not speaking, or chairing at a conference can impact negatively on career development. There is also a welcomed brief reflection on what can be done to encourage a greater gender balance throughout these types of conferences. 

 The following comments could be taken into consideration for further research in this field. Gender equality is much more than ‘equal’ representation of women/ men in terms of participation. For example, the European Commission has since 2012 recognised that gender equality in research and innovation has three main dimensions, 1) careers, (i.e. the representation of women at different stages of careers and in different fields) 2) decision-making and 3) including the gender dimension into research content. Further research in this field might look at how different speakers/ presenters include the gender dimension into their research. Whilst there is a recognition that “ *the proportion of women involved in lecture, workshop, or panel session varied by topic of the session*" – this is underexplored. One way to take this further in future research may be to look at the gender of participants and then examine how this relates to integration of the gender dimension into research content. This could be examined by a scan of title/ abstract.

Is the work clearly and accurately presented and does it cite the current literature?

Yes

If applicable, is the statistical analysis and its interpretation appropriate?

Yes

Are all the source data underlying the results available to ensure full reproducibility?

Yes

Is the study design appropriate and is the work technically sound?

Yes

Are the conclusions drawn adequately supported by the results?

Yes

Are sufficient details of methods and analysis provided to allow replication by others?

Yes

Reviewer Expertise:

Gender Equality in Research and Innovation

I confirm that I have read this submission and believe that I have an appropriate level of expertise to confirm that it is of an acceptable scientific standard.

# Peer review: Reviewer response for version 1

**DOI:** 10.21956/wellcomeopenres.20448.r53822

**Published:** 2023-01-27

**Authors:** Christine Kremer

**Affiliations:** 1 Neurology Department, Skåne University Hospital, Department of Clinical Sciences, Lund University, Malmö, Sweden

In this important article Gibson et al. described the change in the gender distribution at the Royal College of Radiologists` Annual Scientific meeting 2014-2021. They concluded that the proportion of women presenters and keynote speaker reflects that of RCR membership, but not of the patient population.

 Looking more closely at the gender distributions there was an increase from 28.6% of roles held by women in 2014 compared to 46.2% in 2021, which was pronounced during the COVID19 pandemic by OR 1.49. Women were more likely to present a poster than being a speaker or part of a workshop faculty. Regarding the topic of the sessions, women made up one-third up to two-thirds in just 1/15 sessions on interventional radiology. This reflects the ongoing traditional role distribution in the different sub-specialties. One other obvious imbalance is shown by the distribution of the role of keynote speakers, just one third were women between 2014 and 2021. During three years there were not any female keynote speakers, and only one in 2020. One other interesting finding was that the sessions led by women had more women speakers. Conference committee members were predominantly men.

 The article is very well written and structured and covers the main purpose and answers the research questions. The statistical methods used are appropriate.

 I have some points:

- The conclusion in the abstract does not refer to the data presented in the results? How is the distribution in RCR membership? (this is mentioned in the discussion) and which patient population? One often refers to female representation in RCRs compared to patient population which would be something else. There was an increase in roles held by women over time which is not mentioned in the conclusion.
- In the discussion the possible downsides of the pandemic with more women being forced to stay at home with a relative overload of unpaid domestic work might be mentioned. The ones registered to online conferences do not reflect the ones actually being online.
- More pro-active strategies in recruiting women for prestigious roles could be mentioned, like professional or societal networks supporting and promoting women, or analyzing the reasons why more women tend to say no to invitations.
- In summary, these data can serve as source for more directed future efforts to promote gender balance and diversity within medicine and needs to be public. One would like to see more data published, including future follow-ups of the gender distributions throughout conferences and meetings.

Is the work clearly and accurately presented and does it cite the current literature?

Yes

If applicable, is the statistical analysis and its interpretation appropriate?

Yes

Are all the source data underlying the results available to ensure full reproducibility?

Yes

Is the study design appropriate and is the work technically sound?

Yes

Are the conclusions drawn adequately supported by the results?

Yes

Are sufficient details of methods and analysis provided to allow replication by others?

Yes

Reviewer Expertise:

Sex and gender differences in stroke.

I confirm that I have read this submission and believe that I have an appropriate level of expertise to confirm that it is of an acceptable scientific standard, however I have significant reservations, as outlined above.
